# Peroxynitrite induced signaling pathways in plant response to non-proteinogenic amino acids

**DOI:** 10.1007/s00425-020-03411-4

**Published:** 2020-06-13

**Authors:** Pawel Staszek, Agnieszka Gniazdowska

**Affiliations:** Department of Plant Physiology, Institute of Biology, Warsaw University of Life Sciences-SGGW, Nowoursynowska 159, 02-776 Warsaw, Poland

**Keywords:** Canavanine, *meta*-tyrosine, 8-Nitro-guanine, Reactive nitrogen species, Reactive oxygen species, Protein nitration

## Abstract

**Main conclusion:**

Nitro/oxidative modifications of proteins and RNA nitration resulted from altered peroxynitrite generation are elements of the indirect mode of action of canavanine and *meta*-tyrosine in plants

**Abstract:**

Environmental conditions and stresses, including supplementation with toxic compounds, are known to impair reactive oxygen (ROS) and reactive nitrogen species (RNS) homeostasis, leading to modification in production of oxidized and nitrated derivatives. The role of nitrated and/or oxidized biotargets differs depending on the stress factors and developmental stage of plants. Canavanine (CAN) and *meta*-tyrosine (*m*-Tyr) are non-proteinogenic amino acids (NPAAs). CAN, the structural analog of arginine, is found mostly in seeds of Fabaceae species, as a storage form of nitrogen. In mammalian cells, CAN is used as an anticancer agent due to its inhibitory action on nitric oxide synthesis. *m*-Tyr is a structural analogue of phenylalanine and an allelochemical found in root exudates of fescues. In animals, *m*-Tyr is recognized as a marker of oxidative stress. Supplementation of plants with CAN or *m*-Tyr modify ROS and RNS metabolism. Over the last few years of our research, we have collected the complex data on ROS and RNS metabolism in tomato (*Solanum lycopersicum* L.) plants exposed to CAN or *m*-Tyr. In addition, we have shown the level of nitrated RNA (8-Nitro-guanine) in roots of seedlings, stressed by the tested NPAAs. In this review, we describe the model of CAN and *m*-Tyr mode of action in plants based on modifications of signaling pathways induced by ROS/RNS with a special focus on peroxynitrite induced RNA and protein modifications.

**Electronic supplementary material:**

The online version of this article (10.1007/s00425-020-03411-4) contains supplementary material, which is available to authorized users.

## Non-proteinogenic amino acids: canavanine and *meta*-tyrosine

Proteins are synthesized from 20 (plus selenocysteine and pyrolysine) canonical, proteinogenic amino acids (Hendrickson et al. 2004). It is estimated that around 1000 non-proteinogenic amino acids (NPAAs) occur in nature; most of them are of plant or microbial origin (Bell 2003; Vranova et al. 2011; Rodgers 2014). NPAAs play various roles in animals and plants: they are agents of the cellular signaling network, structural components of cell membranes and metabolic intermediates. They also participate in ecological interactions by acting as feeding deterrents or allelochemicals. Many NPAAs seems to be toxic for living organisms: plants, animals, and humans (Rodrigues-Corrêa and Fett-Neto 2019). They are suspected to contribute to serious diseases (e.g. neurodegenerative disease) of unknown etiology (Rodgers 2014). Some of the hundreds of naturally occurring NPAAs, mostly synthesized in plants, are poisoning or can cause clinical disorders. β-*N*-Oxalyl-α,β-diaminopropionic acid, a NPAA present in seeds of grass pea (*Lathyrus sativus* L.), initiates neurolathyrism in humans and some animals (e.g. horses) (Van Moorhem et al. 2011). Leaves and seeds of leucanea (*Leucanea leucophala* Lam. de Witt), a leguminosae tree, contain mimosine (Crawford et al. 2015). This NPAA acts as a chelator of transition metals and its uptake by non-ruminant animals leads to alopecia (fur loss) (Sethi and Kulkarni 1995; Crawford et al. 2015). On the other hand, NPAAs have also beneficial effects as e.g. anti-cancer agents (Rubenstein 2000; Nunn et al. 2010).

**Fig. 1:**
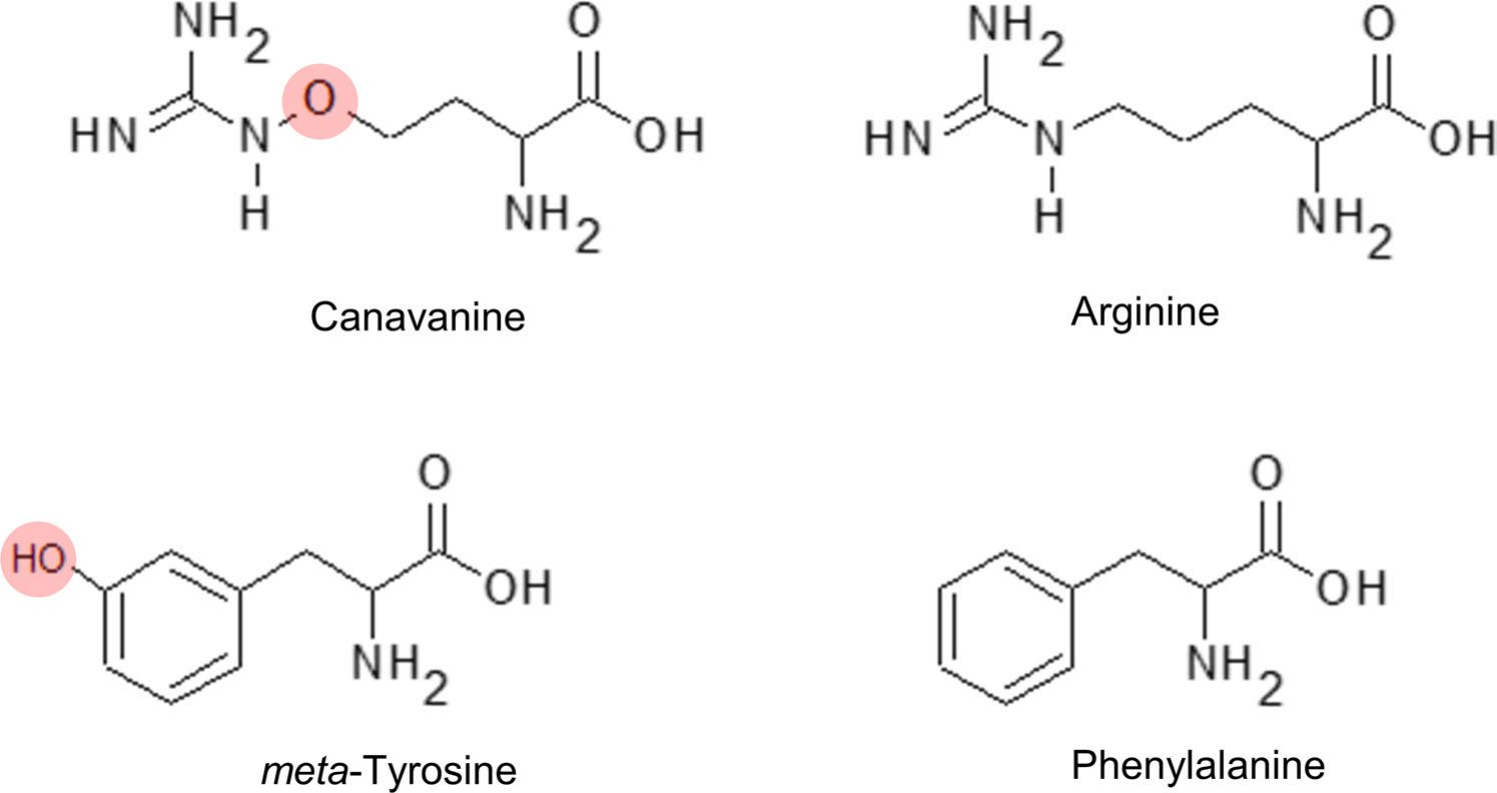
Structure of canavanine (CAN)-analogue of arginine and *meta*-tyrosine (*m*-Tyr)-analogue of phenylalanine

Canavanine (CAN, *L*-2-amino-4-guanidooxy-butanoic acid) belongs to the group of NPAAs which are synthesized in plants. CAN production is limited to some Fabaceae species and it is found mostly in seeds of e.g. jack bean (*Canavalia ensiformis* (L.) DC.), eskimo potato (*Hedysarum alpinum* L.), tropical woody vine (*Dioclea megacarpa* Rolfe) or seeds and sprouts of alfalfa (*Medicago sativa* L.) (Rosenthal 2001), in which it acts as the source of nitrogen. CAN is also an effective toxin that protects plants against herbivores, especially seeds predators. This NPAA is a structural analogue of arginine (Fig. 1), thus can serve as a substrate in every enzymatic reaction that is arginine-dependent. It is commonly accepted that the primary mode of action of CAN and its poisonous effect on living organisms is due to misincorporation into proteins in the place of arginine because arginyl-tRNA synthetase readily esterifies CAN to the cognate tRNA^Arg^ (Rosenthal 2001; Nunn et al. 2010). Insects fed with CAN have been found to synthesize proteins of altered conformation and impaired function (Staszek et al. 2017 and references herein). Introduction of CAN into the diet of tobacco hornworm larvae (*Manduca sexta*) increased their mortality, inhibited growth and resulted in the formation of abnormal adults. But there are still increasing data that action of this NPAA is more complex eg. CAN may be converted by arginase into urea and toxic canaline (Staszek et al. 2017 and references herein). In mammalian, particularly in cancer cells CAN is used to lower nitric oxide (NO) level due to its inhibitory action towards inducible isoform of NO synthase (iNOS) responsible for NO generation from arginine (Kosenkova et al. 2010).

*meta*-tyrosine (*m*-Tyr, L-3-hydroxyphenylalanine) is a structural analogue of proteinogenic amino acid-phenylalanine (Bertin et al. 2007) (Fig. 1). This NPPA is released into the environment by fine-leaf fescue grasses (e.g. *Festuca rubra* spp. Rubra or *F. rubra* spp. Commutata) as root exudates, which make fescues successful competitors to neighboring plants (Bertin et al. 2003). As a strong allelochemical *m*-Tyr was shown to be toxic to a wide range of plant species (Bertin et al. 2003, 2007, 2009). Its primary mode of action similar to CAN is suggested to be incorporated into proteins in place of phenylalanine. Mammalian or microbial phenylalanyl-tRNA synthetase esterifies *m*-Tyr to the tRNA^Phe^ resulting in the synthesis of atypical proteins (Bullwinkle et al. 2014). The synthesis of *m*-Tyr in fescues is based on hydroxylation of phenylalanine (Huang et al. 2012), while in another plant producer of this NPAA-donkey-tail spurge (*Euphorbia myrsinites* L.), it is a product of *m*-hydroxyphenylpyruvate transamination (Huang et al. 2012). Huang et al. (2012) demonstrated that *m*-Tyr is also a product of a non-enzymatic oxidation of phenyalanine by hydroxyl radicals. Thus, in animal cells *m*-Tyr is considered as a marker of oxidative stress and aging (Matayatsuk et al. 2007). Increased level of this NPAA is typical for patients suffering from neurodegenerative diseases such as Alzheimer, a progression of which is linked to reactive oxygen species (ROS) overproduction and disturbances in reactive nitrogen species (RNS) metabolism (Hannibal 2016).

## Peroxynitrite (ONOO^−^) as a key nitrating agent of proteins and nucleic acids

**Fig. 2:**
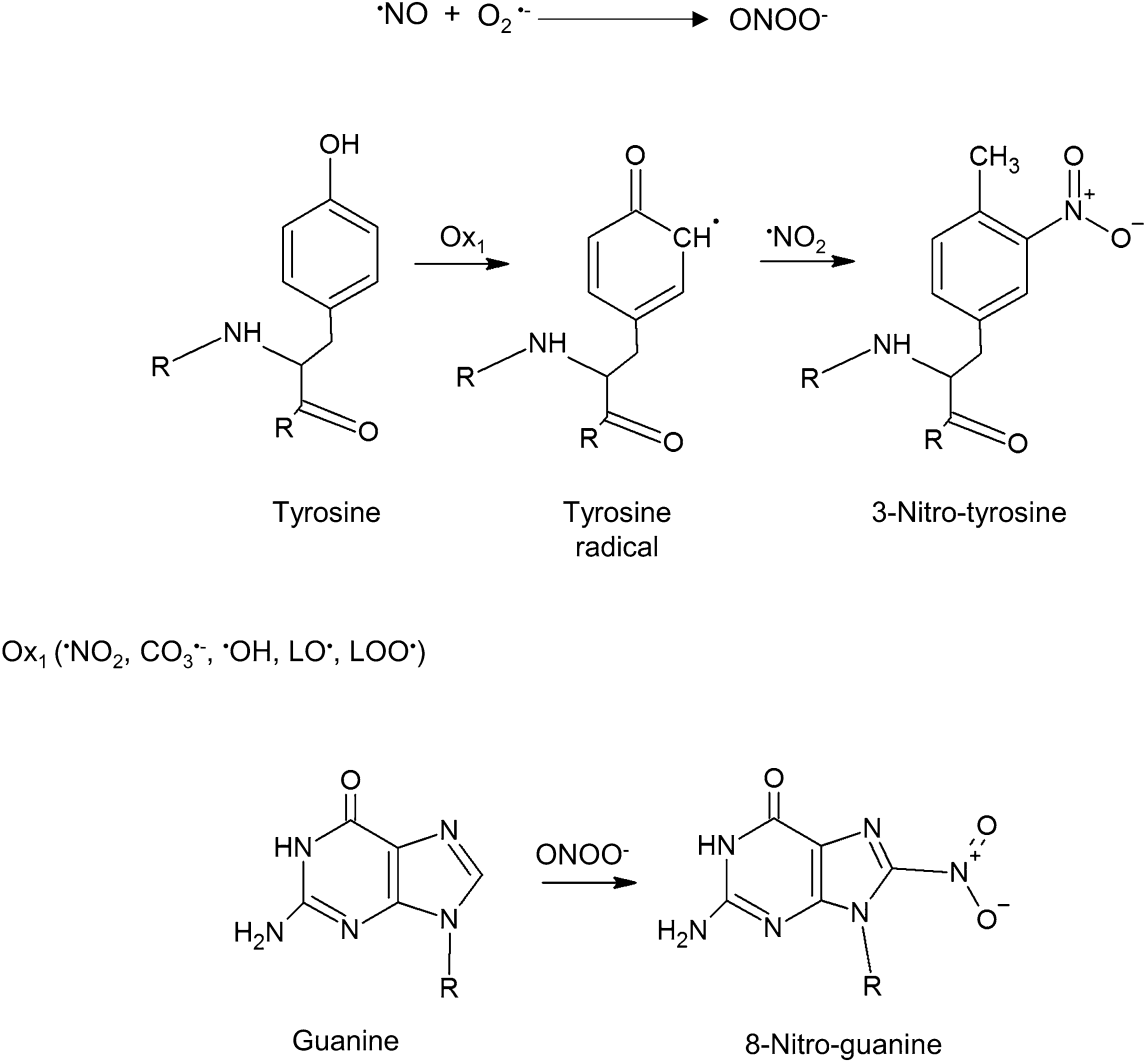
Peroxynitrite (ONOO^−^) formation from NO and superoxide anion (O_2_^•−^); main targets of nitration in plant cells: formation of 3-Nitro-tyrosine (3-NT) and 8-Nitro-guanine (8-NO_2_-G), according to Bartesaghi and Radi (2018) with modification

Nitric oxide (NO), the member of the family of RNS, is a free radical formed in vivo in cells of plants and animals, even though biosynthetic pathways of this molecules in plants and animals are quite different (Kolbert et al. 2019). The story of NO research in plants and doubts on its generation have been perfectly reviewed just recently (Santolini et al. 2017; Del Castello et al. 2019; Kolbert et al. 2019). The action of NO as a signaling molecule or cytotoxic agent depends on its concentration and the redox state of the cell or the cellular compartment. Lamotte et al. (2006) demonstrated that NO regulated cytosolic Ca^2+^ homeostasis in tobacco (*Nicotiana plumbaginifolia* Viv.) cells under hyperosmotic stress by activation of Ca^2+^ channels via signaling cascade involving plasma membrane depolarization, cADP-ribose, and protein kinases. Cytoprotective role of NO, due to activation of the antioxidant system was shown in plants under biotic and abiotic stresses as recently review by Nabi et al. (2019) and Arasimowicz-Jelonek and Floryszak Wieczorek (2016). In the past, the toxicity of NO to living organisms was linked mostly to antropogenic pollution, but nowadays it is investigated in the context of harmful modification of biomolecules (proteins, nucleic acids and lipids) (Begara-Morales et al. 2016). It is important to underline, that NO is not generated in cells independently, it is often produced in stress conditions and is accumulated at the same time as other signaling compounds such as ROS (Hancock and Neill 2019). NO toxicity in the context of oxidative stress conditions is mostly due to the formation of NO-derived oxidants, which are further more reactive than NO itself (Bartesaghi and Radi 2018). NO undergo autooxidation reactions in the presence of O_2_, leading to the formation of nitrogen dioxide (^•^NO_2_)-a strong oxidizing and nitrating agent, although, under normal conditions this process is rather slow. Superoxide radical (O_2_^•−^), a representative of ROS, is regularly formed in cells as a product of oxygen metabolism in the same compartments as NO (Janků et al. 2019). The fast reaction of NO with O_2_^•−^ leads to the formation of peroxynitrite (ONOO^−^), a powerful oxidant, which promotes oxidation and nitration of key cellular molecules: proteins, lipids, and oligonucleotides (Arasimowicz-Jelonek and Floryszak-Wieczorek 2019). This RNS under physiological conditions reacts with CO_2_ and later on is decomposed into CO_3_^−^ and ^•^NO_2_ (Bartesaghi and Radi, 2018). Thus, NO and NO-derived molecules can cause post-translational modifications (PTMs) of target proteins (Mata-Pérez et al. 2016). Protein tyrosine (Tyr) nitration, which is a covalent modification resulting from the addition of a nitro (–NO_2_) group onto one of the two equivalent ortho carbons in the aromatic ring of Tyr, leading to the formation of 3-Nitro-tyrosine (3-NT) is one of the important NO-dependent PTM (Fig. 2) (Kolbert et al. 2017). In contrast to *S*-nitrosylation, Tyr nitration is an irreversible process, highly selective, and of a low yield. Usually, in the whole tissue/cell only 1–5 over 10,000 Tyr residues become nitrated (Bartesaghi and Radi 2018 and references herein). It is suggested that nitration could act as NO-dependent mechanism of regulation of plant metabolism (Mata-Pérez et al. 2016). Beside proteins, as was mentioned, other biomolecules are also targets of nitration. ONOO^−^ reacts with the DNA and RNA bases of guanine (guanine, guanosine and 2′-deoxyguanosine) producing 8-Oxo-guanine (8-oxo-G) and 8-Nitro-guanine (8-NO_2_-G) (Ohshima et al. 2006; Arasimowicz-Jelonek and Floryszak-Wieczorek 2019 and references herein; Chmielowska-Bąk et al. 2019) (Fig. 2). 8-oxo-G mispairs with adenine and cytosine (C), leading to GC → AT, GC → TA, and GC → CG base-pair substitutions (Jena and Mishra 2007). In mammalian cells, these mutations are cancer inducers. Nitration of nucleic acids (DNA and RNA) may be measured as the formation of 8-NO_2_-G, which is relatively stable in the absence of oxidizing agents and as an aqueous solution can be stored at 4 ℃ for several months (Ohshima et al. 2006). Guanine nitration in vivo in biological systems was demonstrated both in animals and plants by immunochemical studies using anti-8-NO_2_-G antibody (Akuta et al. 2006; Izbiańska et al. 2018; Andryka-Dudek et al. 2019 and references herein). Under microbial infections, the time profile of 8-NO_2_-G formation was correlated with the production of NO and 3-NT (Akuta et al. 2006). Due to mechanisms of biosynthesis based on close relation of 8-NO_2_-G to ONOO^−^, 8-NO_2_-G, (alike 3-NT) could be considered as a marker of nitrosative stress. After detection of nitrated nucleic acids using anti-8-NO_2_-G antibodies the elevated level of 8-NO_2_-G in mammalian tissues was demonstrated under chronic inflammation, in cells exposed to air pollutants or cigarette smoke (Ohshima et al. 2006 and references herein). DNA 8-NO_2_ residues may be rapidly depurinated from DNA in vitro, with a half life of 1–4 h under physiological conditions resulting in the formation of mutagenic abasic sites and release of free 8-NO_2_-G (Ohshima et al. 2006). Thus, 8-NO_2_-G in DNA may be potentially mutagenic yielding G:C to T:A transversion. 8-NO_2_-G in RNA is more stable than in DNA (Masuda et al. 2002). 8-NO_2_-G incorporated into RNA may alter RNA function and metabolism. Moreover, as immunoreactivity of 8-NO_2_-G in inflammatory animal cells was detected not only in nucleus but also in cytoplasm and mitochondria; 8-NO_2_-G in nucleotide pool can effect GTP binding proteins, cGMP-dependent enzymes activity and finally modify cell signalling. It is suggested that nitrative and oxidative nucleic acids damage induces not only mutations but also genomic instability and epigenetic changes. Although there are some reports on nitrated DNA or RNA in animal cells, particularly in the context of pathogenesis and carcinogenesis (Murata et al. 2012 and references herein), the data on the nucleic acids nitration in plant cells are unique (Izbiańska et al. 2018; Andryka-Dudek et al. 2019).

Environmental conditions and stresses, including supplementation with toxic compounds, are known to impair ROS and RNS homeostasis, leading to modification in production of oxidized and nitrated derivatives. The role of nitrated and/or oxidized biotargets differ depending on the stress factors and developmental stage of plants. Over the last few years of our research, we have collected the complex data on ROS and RNS metabolism in tomato (*Solanum lycopersicum* L.) plants supplemented with CAN or *m*-Tyr. Therefore, we have made an attempt to show the model of CAN and *m*-Tyr mode of action based on putative signaling pathways induced by ROS and RNS, particularly ONOO^−^ acting as a main nitrosative agent of biomolecules.

## The model of CAN and *m*-Tyr mode of action in plants

**Fig. 3:**
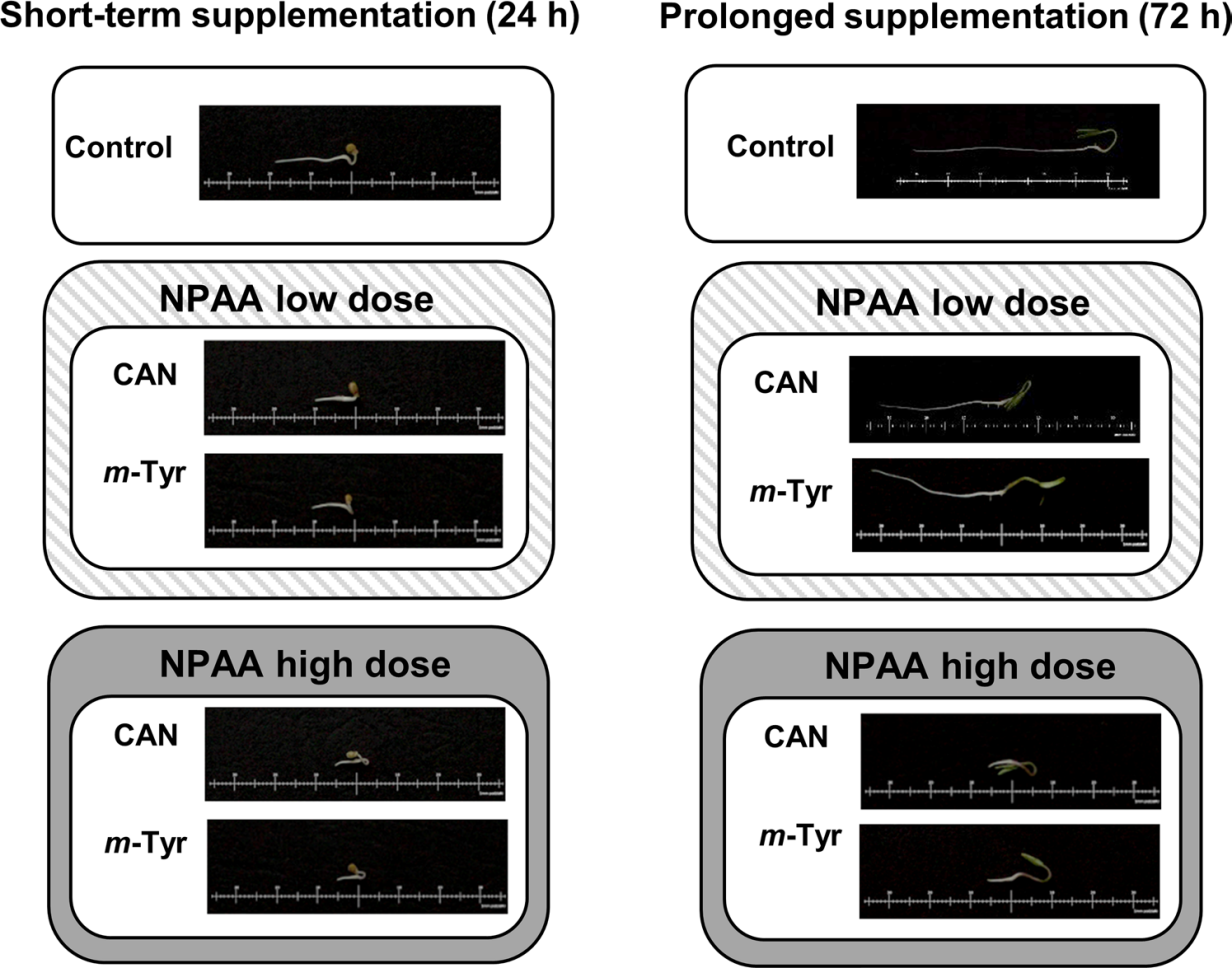
Morphology of the tomato seedlings after 24 or 72 h of supplementation with CAN (10 µM-low dose or, 50 µM-high dose) and *m*-Tyr (50 µM-low dose, 250 µM-high dose)

CAN and *m*-Tyr are classified as the strong toxins, with the primary mode of action based on the formation of aberrant proteins due to their misincorporation into proteins instead of canonical amino acids (Rosenthal 2001; Gurer-Orhan et al. 2006). Although this mode of action of CAN and *m*-Tyr is indisputable, their other indirect activity in animal and plant cells requires further examinations. The first visible morphological effect of CAN and *m*-Tyr supplementation was inhibition of elongation growth of roots of young tomato seedlings (Fig. 3). Root tissue was more sensitive to CAN than to *m*-Tyr because the concentration of the tested NPAAs required for inhibition of roots growth in 50 and 100% was 10 and 50 µM for CAN while five times higher for *m*-Tyr; 50 and 250 µM, respectively (Fig. 3) (Krasuska et al. 2016a, 2017). Negative impact of CAN and *m*-Tyr was limited to roots, while the growth of the shoots of tomato seedlings was not influenced by tested NPAAs (Fig. 3). Similar pattern of physiological toxicity in tomato, maize (*Zea mays* L.) and onion (*Allium cepa* L.) was observed after application of cyanamide (Soltys et al. 2011, 2012, 2014), an allelochemical produced by *Vicia* species and originated in planta from the enzymatic conversion of CAN (Kamo et al. 2015). It could be suggested that the reaction of roots to non-nutritional elements in the soil is plant's defense strategy to minimize uptake of the pollutant.

**Fig. 4:**
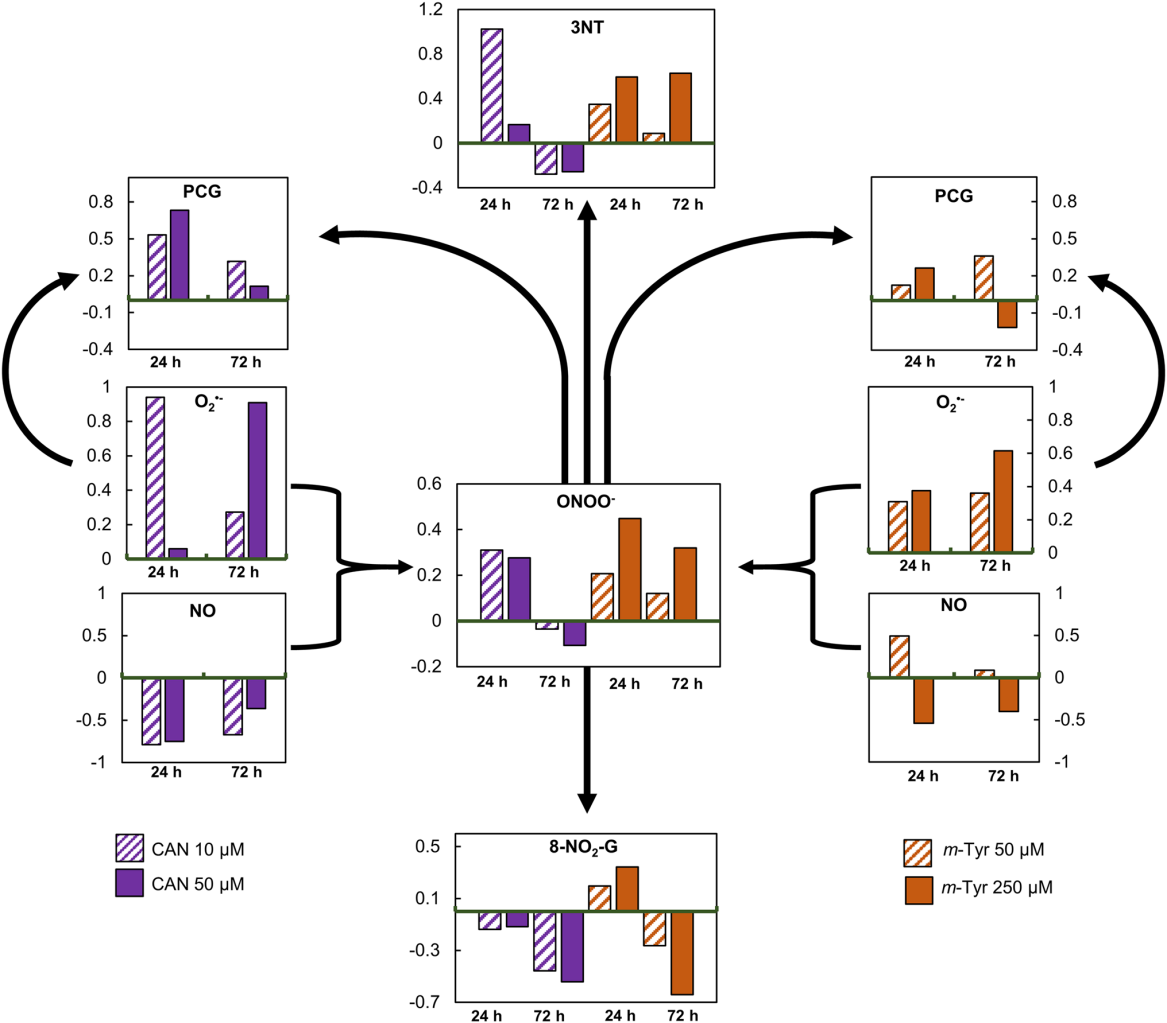
The level of ROS (O_2_^•−^), RNS (NO, ONOO^−^), post translationally modified proteins: nitrated proteins (3-NT) and carbonylated proteins (protein carbonyl groups, PCG) and nitrated RNA (expressed as 8-NO_2_-G) in roots of tomato seedlings supplemented for 24 or 72 h with CAN or *m*-Tyr. Data at the charts are based on data presented by Krasuska et al. (2016a, b), Andrzejczak et al. (2018), converted and expressed as relative units with control at the baseline (green)

Induction of oxidative stress is a typical secondary response of plants to biotic and abiotic environmental factors (Mittler 2002; Gill and Tuteja 2010; Hasanuzzaman et al. 2012) including supplementation with plant originated chemicals and allelochemicals (Gniazdowska et al. 2015). CAN and *m*-Tyr at high or low doses stimulated overproduction and raised the level of ROS (H_2_O_2_ and O_2_^•−^) in roots of tomato seedlings, irrespectively of the duration of the culture (Fig. 4) (Krasuska et al. 2016a, b, 2017; Andrzejczak et al. 2018; Staszek et al. 2019). Disturbances in cellular redox status led to the elevated generation of protein carbonyl groups (Fig. 4) (with the exception of 72 h long treatment with 250 µM m-Tyr) (Krasuska et al. 2016b; Andrzejczak et al. 2018), confirming protein oxidation to be a stable marker of oxidative stress. It could be suspected that the elements of the cellular antioxidant system may be found among oxidised proteins. Such observation was done by Kristensen et al. (2004) in rice (*Oriza sativa* L.) leaves. Analysis of enzymatic antioxidant activity in tomato plants subjected to CAN or *m*-Tyr has shown only slight modification at an enzyme activity level (Andrzejczak et al. 2018; Staszek et al. 2019), whereas transcriptomic approach indicated more spectacular changes (Supplementary material, Table 1). In roots of CAN treated tomato seedlings, activity of main enzymatic antioxidants (catalase and superoxide dismutase) decreased, while glutathione peroxidase (GPx) and glutathione reductase (GR) were generally unaffected (Staszek et al. 2019), despite the expression of GR and GPx after 24 h (Supplementary material, Table 2). In the NPAA fed plants, antioxidant capacity sufficient to scavenge destructive ROS could be maintained rather by non-enzymatic low molecular antioxidants such as phenolics and thiols, level of which increased in roots supplemented with CAN or *m*-Tyr (Andrzejczak et al. 2018; Staszek et al. 2019). Alterations in production of RNS in CAN supplemented seedlings exhibited varied pattern depending on the duration of the experiment; while RNS generation in plants exposed to *m*-Tyr differed depending on the dosage (Fig. 4). The profile of the changes in RNS level in plants grown in CAN or *m*-Tyr is linked mostly to the chemical structure of the NPAAs. CAN (analog of arginine) acts as an inhibitor of arginine dependent NOS-like activity (Staszek et al. 2019), resulting in a deep drop of NO content, and after a longer experiment in limitation of ONOO^−^. ONOO^−^ generation (Fig. 4) in roots of plants treated with *m*-Tyr seems to be regulated rather by the rate of O_2_^•−^ production than NO level, since the dose and time-dependent pattern of changes of the level of both molecules is similar (Fig. 4). Therefore it is not a surprise that alterations in the amount of 3-NT correspond to changes in ONOO^−^, which acts as a main nitrosative agent. Prolonged CAN supplementation resulted in a reduction of protein nitration (Krasuska et al. 2016b), while extended application of 250 µM *m*-Tyr increased 3-NT content in tomato roots (Krasuska et al. 2017) (Fig. 4). This PTM usually leads to conformational alterations, loss of proteins function, inhibition of enzymatic activity, or even facilitates protein degradation (Hancock and Neill 2019; Arasimowicz-Jelonek and Floryszak-Wieczorek 2019). Enzymatic antioxidants were found among the putative targets of nitration in various plants and tissues (Mata-Pérez et al. 2016), therefore tyrosine nitration may alter other reactive signals, particularly ROS signaling. Identification of differentially nitrated proteins in roots of tomato supplemented with CAN for 72 h pointed at monodehydroascorbate reductase (MDAR)-one of the enzymes of Foyer-Halliwell-Asada cycle, which activity was significantly reduced (Staszek et al. 2019).

The nitration process is specifically involved in various cell regulatory mechanisms and new evidence shows that nitrative modifications of nucleic acids can be considered also as a regulator of gene expression. RNA nitration level in roots of tomato seedlings was measured as 8-NO_2_-G content in total RNA (supplementary material). In roots of control seedlings, RNA nitration was constant during the experiment (Table 1). In seedlings grown with NPAAs, RNA nitration level decreased as the culture was prolonged. Plants supplementation with CAN for 24 h did not change the content of 8-NO_2_-G in comparison to the seedlings grown in water, while 48 h longer exposition of the seedlings to this NPAA resulted in decreased 8-NO_2_-G content to about 50% of the control, independently of the dose (Table 1). Application of *m*-Tyr for 24 h led to increased RNA nitration level in roots. Prolongation of the feeding of the seedlings with *m*-Tyr resulted in lower 8-NO_2_-G level, which was particularly evident after application of *m*-Tyr at the higher dose (Table 1). In animal tissue formation of 8-NO_2_-G correlated with the production of NO and 3-NT under microbial infections (Akuta et al. 2006). The pattern of changes in 8-NO_2_-G in RNA after tomato plants supplementation with CAN or *m*-Tyr (Fig. 4) did not exactly match the pattern of 3-NT amount nor the profile of the changes in ONOO^−^ level, except for seedlings treated with 50 µM *m*-Tyr for 24 h. There may be at least two explanations of this observation: (1) 8-NO_2_-G can be converted to 8-oxo-G and other 8-oxopurines in further reaction with ONOO^−^. (2) Jena and Misra (2007) demonstrated that formation of 8-oxo-G in the reaction of ONOO^−^ with guanine is probably preferred over that of the 8-NO_2_-G. Therefore, the appearance of 8-oxo-G may be predominant. Thus, in future experiments, it would be interesting to measure the level of 8-oxo-G.

**Table 1:** RNA nitration level (8-NO_2_-G, ng μg-1RNA) in tomato roots after 24 or 72 h of seedlings supplementation with CAN (10, 50 μM) or *m*-Tyr (50, 250 μM). Control plants were grown in water

Supplementation	24 h	72 h
control	62.2±4.3	72.7±10.2
CAN 10 μM	53.7±11.0	39.5±6.9*
CAN 50 μM	55.0±5.6	33.3±14.1*
*m*-Tyr 50 μM	74.5±1.0*	53.7±9.2
*m*-Tyr 250 μM	83.6±8.6*	26.1±4.5*

Two technical replicates were performed for each three-four biological replicates

Mean values ± SD, asterisks (*) indicate difference from the control

In mammalian cells, the formation of 8-NO_2_-G initiates different types of mutation, inflammation, and cancers. It was suggested that the 8-NO_2_-G lession can cause G to T mutation by either mispairing with A or through depurination to yield apurinic sites (Bhamra et al. 2012). Although it is still unclear which of the mechanisms is more important. Even if there is no doubt that in animal cells 8-NO_2_-G is characteristic for pathogenesis, its role in plants is unclear. Data presented in this work (Table 1) are the third report on the determination of 8-NO_2_-G in RNA in plants. Izbiańska et al. (2018) have shown a transient increase of 8-NO_2_-G in the RNA and mRNA pools in potato leaves after infection with *Phytophtora infestans*. They proposed that in plants formation of 8-NO_2_-G may be a selective modification, regulating the post-transcriptional gene expression and participating in cell signaling resulting in active cell death. Increased total RNA nitration level was detected also in axes of apple embryos during the transition from a dormant to a non-dormant state (Andryka-Dudek et al. 2019). It was suggested that modification of DNA or RNA could be of great importance in signaling pathways of ROS and RNS both in developmental processes and in plant reactions to environmental stimuli, similarly as it was proposed for oxidized mRNA. Bazin et al. (2011) demonstrated not only the presence of RNA oxidation during after-ripening of sunflower (*Helianthus annuus* L.) seeds but also linked it to artifacts in cDNA and alterations in protein translation. They identified specified stored mRNAs highly oxidized which corresponded to genes involved in responses to stress and in cell signaling. It can be suspected that RNA nitration in plants supplemented with toxic NPAAs as the accumulation of oxidized transcripts in response to environmental stressors e.g. cadmium (Chmielowska-Bąk et al. 2017) may cause ribosome stalling, and in consequence slow down the translation (Nunomura et al. 2017). In short term experiment in roots of *m*-Tyr supplemented tomato seedlings, 8-NO_2_-G level was higher than in the control plants (Table 1, Fig. 4), therefore we could expect the decrease of the number of encoded proteins. Moreover, differentially nitrated transcripts encoding e.g. elements of antioxidant cellular system could explain alterations in their transcription after application of CAN (Staszek et al. 2019) or *m*-Tyr (Andrzejczak et al. 2018) (supplementary material, Table 1 and Table 2) and finally the total activity of the antioxidant system. In addition, it was shown that 8-NO_2_-G generated in the viral genome due to overproduction of NO resulted in increased frequency of mutation of an RNA virus (Ihara et al. 2011 and references herein). Thus elevated 8-NO_2_-G in RNA of *m*-Tyr supplemented tomato plants may point at impaired RNA function, although further experiments confirming this assumption should be performed.

Treatment of plants with NO or its donors results in changes at the transcriptomic level (Hussain et al. 2016; Imran et al. 2018; Andryka-Dudek et al. 2019 and references herein). Those changes may be explained by NO-dependent PTMs of proteins e.g. *S*-nitrosylation or nitratrion of transcription factors (Palmieri et al. 2008). But quite recently it was suggested that in Arabidopsis NO donor (*S*-nitrosoglutathione, GSNO) impacts genes transcription by affecting chromatin state via histone acetylation (Mengel et al. 2017). GSNO increased histone acetylation, probably by *S*-nitrosylation and subsequent inhibition of histone deacetylases (HDACs) (Mengel et al. 2017). Global histone hyperacetylation is typical in plants under stress conditions (heat, salt, and cold) (Mengel et al. 2017 and references herein), which are also characterized by the elevated generation of RNS. HDACs are transcriptional repressors of stress responses (Luo et al. 2012; Choi et al. 2012; Zheng et al. 2016), thus the correct response to stress needs the inactivation or elimination of HDACs (Choi et al. 2012). According to the model proposed by Mengel et al. (2017) the inactivation of HDACs during stress perception might be mediated by NO. Stress initiates NO production and NO inhibits HDAC complexes by redox PTMs and finally enhances histone acetylation. It promotes changes in chromatin state, favouring the expression of stress-related genes. Although at that moment there are no data confirming the link between chromatin state and 8-NO_2_-G level both factors at the transcriptomic level may influence plant reaction to supplementation with NPAAs via NO-dependent manner.

In mammalians, 8-NO_2_-guanosine was also found to stimulate the generation of O_2_^•−^ via the uncoupling of NOS isoforms and other reductase-like enzymes e.g. P450 reductase and xanthine oxidase (Sawa et al. 2013). It cannot be excluded that increased O_2_^•−^ level in tomato roots fed with toxic NPAAs could be the product of e.g. stimulation of xantine oxidase, thus 8-NO_2_-G could act as a feedback regulator.

Nitroguanosine 3′,5′-cyclic monophosphate (8-nitro-cGMP) is an endogenous cGMP derivative. Its formation and action were shown in plants in guard cells (Joudoi et al. 2013). 8-Nitro-cGMP reacts with protein sulfhydryls resulting in protein *S*-guanylation. In animal cells, this PTM is involved in the regulation of cellular responses to oxidative, metabolic and environmental stresses (Sawa et al. 2013). Moreover, it was reviewed that intracellular 8-nitro-cGMP formation and 8-NO_2_-G formation in mammalian tissue had similar immunostaining profile depending on the location and duration of the experiment (Ihara et al. 2011). Thus, the putative excess formation of 8-nitro-cGMP in tomato plants supplemented with CAN or *m*-Tyr could be another factor that may influence root growth. One of the important features of this sygnalling molecule (8-nitro-cGMP) is its dependence on cellular production of ROS, which is elevated after NPAAs supplementation. In animals, scavenging of ROS diminished 8-nitro-cGMP almost completely (Ahmed et al. 2012). cGMP plays an important role in the auxin-regulated determination of root morphology, growth and development (Bai et al. 2012; Nan et al. 2014), and its accumulation in roots increased in response to auxin application in a concentration-dependent manner (Pagnussat et al. 2003; Bai et al. 2012; Nan et al. 2014). CAN (Krasuska et al. 2016a) and *m*-Tyr (Olechowicz 2019) led to overaccumulation of auxins, therefore, it can be suspected that malformations in root structure could be in part the result of presumed changes in cGMP and 8-nitro-cGMP level. This hypothesis requires experimental verification because the role of 8-nitro-cGMP in plants cells is still unexplored.

Phenolics are regarded as scavengers of ROS and RNS (Arasimowicz-Jelonek and Floryszak-Wieczorek 2019 and references herein). Phenolic compounds are also important targets of nitration. Monophenols undergo in vitro ONOO^−^- dependent nitration, but it was also shown that *p*-coumaric acid may be nitrated in an ONOO^−^-independent reaction that involves horseradish peroxidase (peroxidase klass III), NaNO_2_ and H_2_O_2_ (Ramezanian et al. 1996; Sakihama et al. 2003). Therefore, as *m*-Tyr at higher dose increased concentration of total phenolics (Andrzejczak et al. 2018), enhanced nitration of phenolic compounds could be expected. Their deposition in the cell wall cannot be excluded, thus *m*-Tyr apart from inhibiting root elongation growth may lead to malformations in root diameter (Krasuska et al. 2017) probably due to the thickening of cell walls. Peroxidase/NO_2_ dependent nitration of phenolic compounds in the apoplastic space provides intermediates for lignin biosynthesis (Sakihama et al. 2003), therefore this could partially explain root abnormalities in *m*-Tyr supplemented plants.

## Summary

**Fig. 5:**
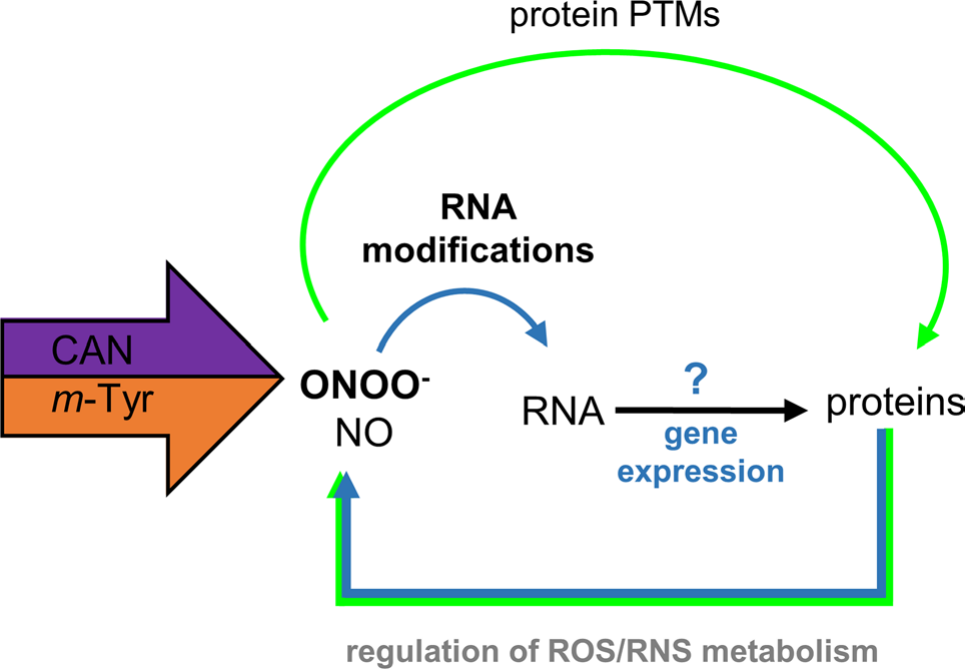
The model of CAN and *m*-Tyr indirect mode of action in root cells. CAN and *m*-Tyr induce disturbances in RNS (ONOO^−^ and NO) level. The main nitrosative agent (ONOO^−^) (generated in cells in response to CAN and *m*-Tyr supplementation) leads to nitration of RNA. Modification in the level of RNA nitration (content of 8-NO_2_-G) may influence gene expression resulting in changes in protein quantity and quality. In addition, ROS/RNS dependent protein PTMs (nitration and/or carbonylation) modify proteins enzymatic activity. Decreased activity of proteins (enzymes of antioxidant cellular system) impacts ROS metabolism/scavenging, that influences ONOO^−^ generation

NPAAs are plant originated chemicals that in the natural environment may have an impact on growth and development of other organisms. In addition to the basic mechanism of their toxicity (misincorporation in proteins instead of canonical amino acids) we demonstrated that harmfulness of CAN or *m-*Tyr is due to an alteration in ROS/RNS metabolism (Fig. 4). ONOO^−^ seems to be the main mediator of the indirect mode of action of CAN and *m*-Tyr (Fig. 5). ONOO- reactions with biomolecules such as proteins, nucleic acids or phenolics lead to oxidative damage (Bartesaghi and Radi 2018), but they can also induce cellular reprogramming. Oxidative and nitrative modifications of proteins were detected in roots of tomato seedlings cultured in the presence of CAN or *m*-Tyr. The alterations (decrease or increase) in the content of total nitrated RNA were also noticed. Therefore, we can conclude that according to the suggestion of Ihara et al. (2011) nitrative nucleotide modifications may be not only a simple chemical damage that leads to a loss of the biological function but may be a physiologically relevant phenomena that allows the cells to evoke signaling for adaptive responses to the chemical stress. We suspect that variation in 8-NO_2_-G content may impact genes expression and could act as a mechanism of plants response to supplementation with toxic compounds (Fig. 5).

### *Author contribution statement*

Conceptualization**:** PS, AG; Formal analysis and investigation: PS; Writing-original draft preparation: PS, AG; Writing-editing and figure preparation: PS, AG; Supervision: AG; Funding acquisition: AG. All authors read and approved the final version of the manuscript.

## Electronic supplementary material

Below is the link to the electronic supplementary material.

Supplementary file1 (PDF 255 kb)

## Abbreviations

3-NT: 3-Nitro-tyrosine
8-nitro-cGMP: Nitroguanosine 3′,5′-cyclic monophosphate
8-NO_2_-G: 8-Nitro-guanine
8-oxo-G: 8-oxo-guanine
CAN: Canavanine
cGMP: Guanosine 3′,5′-cyclic monophosphate
GSNO: *S*-Nitrosoglutathione
HDAC: Histone deacetylase
*m*-Tyr: *meta*-tyrosine
NO: Nitric oxide
NPAA: Non-proteinogenic amino acid
ONOO^−^: Peroxynitrite
PTM: Posttranslational modification
RNS: Reactive nitrogen species
ROS: Reactive oxygen species
