# Supplementary material for: Peroxynitrite induced signaling pathways in plant response to non-proteinogenic amino acids
Source: Planta. 2020 Jun 13;252(1):5. doi: 10.1007/s00425-020-03411-4 (PMC7293691; doi:10.1007/s00425-020-03411-4)
Supplement: Supplementary file 1 — Supplementary file1 (PDF 255 kb) [file 425_2020_3411_MOESM1_ESM.pdf]

Plant material and experimental conditions

Tomato seeds (*Solanum lycopersicum* L. cv. Malinowy Ożarowski) were germinated in water for 3-4 days at 20ºC in darkness. Seedlings with equal roots (5 mm length) were selected and transferred to Petri dishes (Ø 9 cm) lined with filter paper and moistened with water (control) or water solutions of CAN (L-enantiomer, Sigma-Aldrich) or *m*-Tyr (DL-enantiomer, Sigma-Aldrich). The seedlings were cultured in a growth chamber at 12/12 h day/night regime 23/20 ºC for 24 and 72 h as was described (Krasuska et al. 2016).

Seedlings were treated with NPAAs in low dose (10 µM CAN and 50 µM *m*-Tyr), which inhibited root growth in 50 % and in high dose (50 µM CAN and 250 µM *m*-Tyr), which led to total inhibition of root growth without lethal effect (Krasuska et al. 2016, 2017). The scheme of plants treatment with NPAAs is shown at Sup. Fig 1.

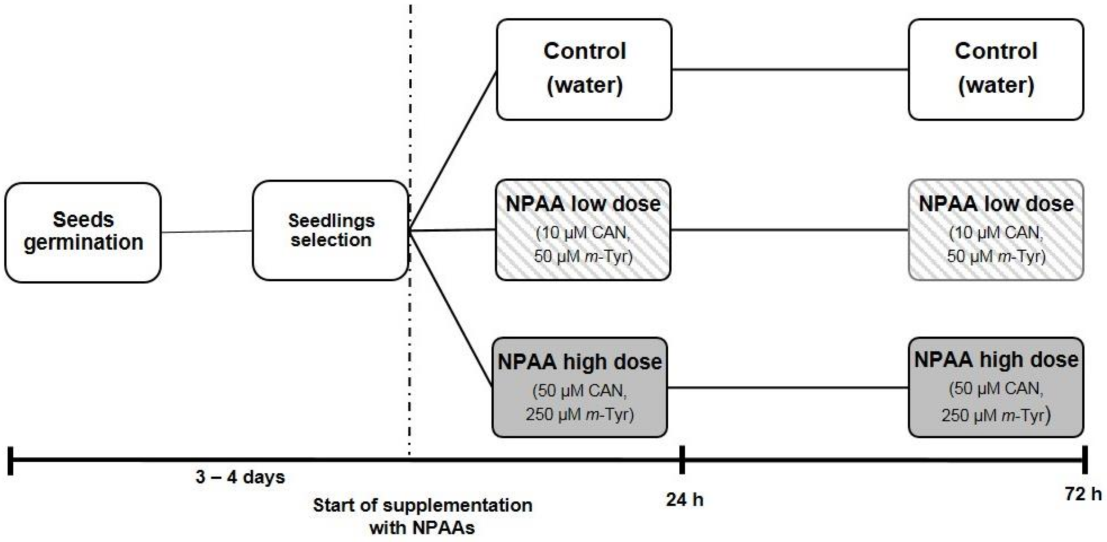

Supplementary Figure. 1. The scheme of the experiment. After 3-4 days of tomato seeds germination in water seedlings were selected based on the length of the roots (5 mm long) and supplemented with CAN or *m*-Tyr. Roots of seedlings supplemented with NPAAs for 24 h or 72 h were used as experimental material.

Measurement of RNA nitration level

For RNA isolation, 400 mg of roots of tomato seedlings (frozen in liquid nitrogen and stored in -80ºC) were homogenized with mortar and pestle with liquid nitrogen. RNA was extracted and purified using RNAzol®RT (Sigma, R4533) according to the manufacturer's instructions.

RNA nitration level was measured as concentration of 8-NO_2-G using OxiSelect™ Nitrosative DNA/RNA Damage ELISA Kit (Cell Biolabs INC., STA-825). 96-wells microplate was coated with 100 µl of 8-nitroguanine-BSA conjugate and incubated at RT for 1 h with gentle shaking on an orbital shaker. Then, the plate was kept overnight at 4° C. After that, the coating solution was discarded and the plate was washed with 1 x PBS and excess liquid was removed with a paper towel. For blocking, 200 µl of assay diluent was used. After 2 h at RT assay diluent was removed and 50 µl of a sample (120-180 µg of total RNA) was added and incubated on an orbital shaker at RT for 10 min. 8-NO_2-G was used as a standard (concentration from 0 ng ml^{-1} to 1000 ng ml^{-1}).

For immunolabelling 50 µl of anti-8-NO_2-G antibody diluted 1:2000 was used. After 1 h of incubation on an orbital shaker at RT, the solution was discarded and the plate was washed 3 times with washing buffer. 100 µl of Secondary Antibody, Horseradish Peroxidase Conjugate (diluted 1:1000) were added and the plate was incubated for 1 h at RT. The solution was removed and the plate was washed 3 times with washing buffer. The enzymatic reaction was started by addition of 100 µl of substrate solution. The reaction was run at RT on an orbital shaker until the color appeared. The enzyme reaction was stopped by adding 100 µl of stop solution. The absorbance was measured on a microplate reader (Sunrise, Tecan) at 450 nm. Nitrated RNA level was calculated using the standard curve. Experiments were carried out in three biological replicates in two technical repetitions.

Image acquisition

Images of seedlings were taken with TAGARNO FHD TREND digital microscope with magnification 5.5x, aperture f/5.6, shutter speed 1/90, enhancement 20.5. Representative images were shown.

Statistical analysis

Data were analyzed using Statistica Software. Mean differences were calculated using t-test, ±SD was also provided to indicate the variations associated with the particular mean values. The published data were converted and expressed in relative units.

References

Krasuska U, Andrzejczak O, Staszek P, et al (2016) Toxicity of canavanine in tomato (*Solanum lycopersicum L.*) roots is due to alterations in RNS, ROS and auxin levels. Plant Physiol Biochem 103:84–95. https://doi.org/10.1016/j.plaphy.2016.03.005

Krasuska U, Andrzejczak O, Staszek P, et al (2017) *meta* -Tyrosine induces modification of reactive nitrogen species level, protein nitration and nitrosoglutathione reductase in tomato roots. Nitric Oxide 68:56–67. https://doi.org/10.1016/j.niox.2016.10.008

Supplementary Table 1. Changes of the expression of genes encoding different isoforms of superoxide dismutase (SOD) and catalase (CAT) in roots of tomato seedlings treated with CAN (10 µM, 50 µM) or *m*-Tyr (50 µM, 250 µM) for 24 or 72 h. Based on data presented by Staszek et al. (2019) and Andrzejczak et al. (2018)

↑ (expression higher than in the control) ↓ (expression lower than in the control) ± (no significant changes in the comparison to the control)

Gene ID (NCBI)	Gene name	CAN 10 µM		CAN 50 µM		Reference	*m*-Tyr 50 µM		*m*-Tyr 250 µM		Reference
		24 h	72 h	24 h	72 h		24 h	72 h	24 h	72 h	
544259	*FeSOD*	↑	↓	±	↓	Staszek et al. (2019)	↑	↑	↑	↑	Andrzejczak et al. (2018)
101256386	*MnSOD*	±	±	↑	↑		±	↓	↓	↓	
101264296	*CuSOD*	±	↓	±	↓		↑	↓	↑	±	
543981	*SODCP-2*	±	±	↑	↓		↑	↓	↓	↓	
101256231	*SOD3*	↓	±	↑	±		↑	↑	↑	↑	
543990	*CAT1*	±	±	↑	↑		↑	↓	↑	↓	
543585	*CAT2*	↓	±	↓	↓		±	↓	↓	↓	
101259333	*CAT3*	↓	↓	↓	↓		±	↓	↓	↓	

Supplementary Table 2. The expression of genes encoding different isoforms of glutathione reductase and glutathione peroxidase in roots of tomato seedlings treated with CAN (10 µM, 50 µM) for 24 or 72 h. Based on data presented by Staszek et al. (2019)

↑ (expression higher than in the control) ↓ (expression lower than in the control) ± (no significant changes in the comparison to the control)

Gene ID (NCBI)	Gene name	CAN 10 µM		CAN 50 µM		Reference
		24 h	72 h	24 h	72 h	
100301931	*GR1*	↓	↓	±	±	Staszek et al. (2019)
100301935	*GR2*	±	±	±	↓	
544197	*GPXle-1*	±	±	↑	±	
101267098	*GPXle-2*	±	±	↑	±	
01267388	*pGPx8*	↓	±	↓	±	
544261	*GPx*	↓	±	±	↑	

References

Andrzejczak O, Krasuska U, Olechowicz J, et al (2018) Destabilization of ROS metabolism in tomato roots as a phytotoxic effect of *meta*-tyrosine. Plant Physiol Biochem 123:369–377. https://doi.org/10.1016/j.plaphy.2017.12.024

Staszek P, Krasuska U, Otulak-Kozieł K, et al (2019) Canavanine induced decrease in NO synthesis alters activity of antioxidant system but does not impact GSNO catabolism in tomato roots. Front Plant Sci 10:1077. https://doi.org/10.3389/FPLS.2019.01077
